# Bleeding management: rFVIIIFc in hemophilia A and liver transplantation

**DOI:** 10.1007/s00101-023-01320-1

**Published:** 2023-08-10

**Authors:** Oliver Grottke, Annette Rieg, Florian Ulmer, Marc Hein

**Affiliations:** 1 Department of Anaesthesiology, RWTH Aachen University Hospital, Pauwelsstraße 30, 52074 Aachen, Germany; 2 Department of Surgery, RWTH Aachen University Hospital, Aachen, Germany

**Keywords:** Hemophilia A, Bleeding management, Recombinant factor VIII, Liver transplantation, Efmoroctocog alfa, Hämophilie A, Blutungsmanagement, Rekombinanter Faktor VIII, Lebertransplantation, Efmoroctocog alfa

## Abstract

**Background:**

In patients with severe hemophilia A prolonged bleeding may occur even in cases of minor trauma or surgery.

**Objective:**

To investigate the feasibility and efficacy of a recombinant extended half-life (EHL) FVIII concentrate for perioperative bleeding management in a patient with severe hemophilia A undergoing liver transplantation.

**Material and methods:**

Prior to transplantation FVIII activity and perioperatively required FVIII supply were estimated. In an individualized approach efmoroctocog alfa was supplemented if the intrinsic clotting time in the thrombelastometry was > 170 s.

**Results:**

The patient perioperatively received a total of 28,000 IU efmoroctocog alfa. No signs of hemorrhage or complications were detected and no further intervention was necessary.

**Conclusion:**

The present case demonstrates that the use of an EHL FVIII is suitable for a successful perioperative bleeding control even in hemophilia patients at a high bleeding risk during major surgery. Due to the EHL constant FVIII levels could be achieved with relatively few injections. In order to confirm the obtained results, more real-world data in different operative settings are essential. Further research is needed on the use of thrombelastometry to guide substitution of factor VIII perioperatively.

## Abstract (de)

**Hintergrund:**

Bei Patienten mit schwerer Hämophilie A können bereits kleine Traumata oder Operationen zu anhaltenden Blutungen führen.

**Ziel der Arbeit:**

Untersuchung der Machbarkeit, Wirksamkeit und Sicherheit von einem rekombinantem halbwertszeitverlängerten FVIII im perioperativen Blutungsmanagement bei einem Patienten mit schwerer Hämophilie A und Lebertransplantation.

**Material und Methoden:**

Vor Transplantation wurden FVIII-Aktivität und erforderliche perioperative FVIII-Gabe geschätzt; in einem individualisierten Vorgehen wurde Efmoroctocog alfa bei Gerinnungszeiten >170 s (InTEM-Test) in der Thrombelastometrie supplementiert.

**Ergebnisse:**

Der Patient erhielt perioperativ kumulativ 28.000 IE Efmoroctocog alfa. Weder Blutungen noch andere Komplikationen traten auf, weitere Interventionen waren nicht erforderlich.

**Diskussion:**

Die Therapie mit einem halbwertszeitverlängerten FVIII war wirksam und sicher zur perioperativen Blutungskontrolle bei einem Patienten mit schwerer Hämophilie A und Lebertransplantation. Aufgrund der verlängerten Halbwertszeit wurden konstante FVIII-Spiegel mit relativ wenigen Injektionen erreicht. Weitere Daten aus dem klinischen Behandlungsalltag sind notwendig, um unsere Ergebnisse zu bestätigen und die Möglichkeit der Anwendung der Thromboelastometrie zur Steuerung der Faktor-VIII-Substitution zu überprüfen.

## Medical history

A 46-year-old man with a history of variceal and other bleeding events as well as B‑cell lymphoma had a diagnosis of severe hemophilia A since early childhood. This inherited bleeding disorder is characterized by recurrent spontaneous bleeding, especially into the joints and muscles as well as severe posttraumatic bleeding with prolonged wound healing due to a deficiency in clotting factor VIII (FVIII) [5]. To date, different recombinant FVIII products are available on the market. In our patient in 2018 treatment was switched from regular prophylaxis of hemophilia A to 4000 international units (IU) of recombinant FVIII Fc fusion protein (rFVIIIFc) efmoroctocog alfa (Elocta®, Sobi-Biogen) twice weekly, equivalent to 106.7 IU/kg at a body weight of 75 kg. The rFVIIIFc with an extended half-life (EHL) compared to standard FVIII concentrates facilitates longer intervals between doses or higher FVIII levels with the same dosing intervals [3] and was well tolerated by the patient.

The patient’s lifetime FVIII replacement therapy from pooled blood donors prior to viral inactivation led to a human immunodeficiency virus (HIV) and hepatitis C virus (HCV) co-infection. The patient received continuous highly active anti-retroviral treatment (HAART) to effectively control the HIV infection. Despite several anti-HCV therapies, virus-related cirrhosis was diagnosed in 2012 and 3 years later the HCV infection was resolved using an antiviral combination therapy.

## Diagnosis

In 2019 the patient developed HCV-related end-stage liver disease with a Child-Pugh status B.

## Therapy and its course

**Fig. 1:**
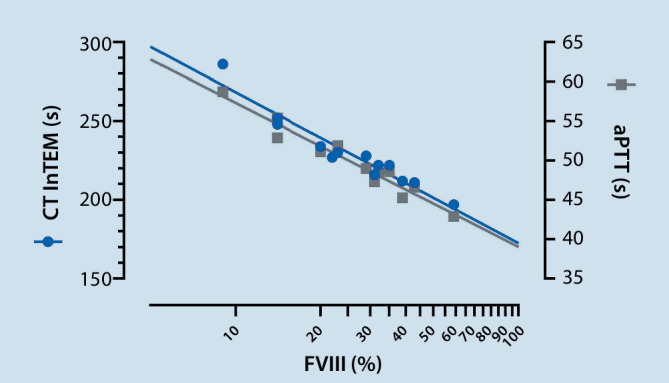
The pharmacokinetic profile of the patient was established prior to surgery. The InTEM clotting time (CT InTEM) and aPTT were measured to predict FVIII activity (FVIII). Depending on the intraoperative results of the CT InTEM, efmoroctocog alfa was substituted accordingly

The patient was scheduled for an orthotopic liver transplantation (OLT), representing the treatment of choice for hemophilia patients with virus-related liver cirrhosis. The preoperative coagulation management strategy for the planned surgical intervention was based on the administration of rFVIIIFc on demand. The World Federation of Hemophilia recommends FVIII levels of at least 80–100% presurgery and of 60–80% for days 1–3 postsurgery [5]. During liver transplantation higher levels were recommended. Before reperfusion, a level of 120% was suggested and after reperfusion 80–100% [6]. Taking these target values into account and having no 24/7 FVIII measurement available, we developed a scheme for the patient to predict FVIII activities based on the intrinsic stimulated thrombelastometry assay (InTEM). Briefly, the effect of the routine substitution of efmoroctocog alfa was monitored by coagulation measurements including InTEM (clotting time, CT), FVIII activity and activated partial thromboplastin time (aPTT). Measurements were made before and 30 min after application of 4000 IU and 3 times after 1000 IU of efmoroctocog alfa at a time interval of 120 min. Based on the results we developed a prediction model to estimate the required perioperative supply of FVIII concentrate. Based on the results, efmoroctocog alfa was supplemented if the clotting time of the InTEM assay was > 170 s and the actual FVIII activity was estimated from the degree of the prolongation (Fig. 1):

$$\text{Predicted FVIII activity }[\mathrm{{\%}}]=10^{((\text{CTINTEM }-364)/-95.7)}$$

The dosage of efmoroctocog alfa was calculated from the observed increase of FVIII activity (0.01 ± 0.002%/IU):

$$\text{FVIII dosage }=(\text{target FVIII activity }-\text{actual FVIII activity }[\mathrm{{\%}}])\cdot 100[\mathrm{IU}]$$

It is important that the formula given, despite its supposed mathematical accuracy, is only a very rough estimate in this individual case in the absence of a timely factor VIII determination. Coagulation markers such as aPTT, FVIII and fibrinogen levels, platelet count (Plt), prothrombin time (PT) and InTEM assay were constantly monitored throughout the transplantation period (Table 1). Fresh frozen plasma (FFP) was transfused continuously during the surgery, red blood cell concentrates (RBC), fibrinogen and platelets were supplied, if required. Preoperative testing revealed a FVIII activity of 19%. While preparing anesthesia, an initial bolus of 8000 IU efmoroctocog alfa was administered followed by a second dose of 4000 IU 1.5 h later. The FVIII activity increased to > 150% 1h before extraction of the cirrhotic liver and start of the anhepatic period and remained constant as determined 90 min later. At the time points 5.5 h, 6.0 h and 7.5 h after transfer to the operating theater and induction of anesthesia, the patient received further doses of 4000 IU efmoroctocog alfa. Predicted FVIII activity (InTEM) revealed a level of 60% at the time of the last bolus injection 7.5 h after OLT start, whereas measurement of FVIII activity revealed a level of 121%; however, InTEM-based clotting time measurement revealed stable values within the normal range of 100–240 s throughout the transplantation period.

**Table 1:** Pharmacokinetic analyses during and after orthotopic liver transplantation

Laboratory analyses	Hours after OLT start									Days postsurgery		
	0 h	1.5 h	2.5 h	3 h	4.5 h	5.5 h^†^	6 h	7.5 h	8.5 h	1 d	2 d	4 d
InTEM CT (s)	215	187	158	155	151	173	208	194	183	n. a.	n. a.	n. a.
Predicted FVIII activity (%)	36	71	142	153	168	99	43	60	80	n. a.	n. a.	n. a.
Target FVIII activity (%)	120	120	120	120	120	120	100	100	80	80	80	80
Efmoroctocog alfa (IU)	8000	4000	–	–	–	4000	4000	4000	–	4000	–	–
Measured FVIII activity (%)	19	n. a.	n. a.	> 150	> 150	n. a.	n. a.	121	n. a.	65	109	61
aPTT (s)	56.9	n. a.	n. a.	30.9	29.1	n. a.	n. a.	34.3	n. a.	33.4	27	30.5
PT (%)	51	n. a.	n. a.	69	71	n. a.	n. a.	70	n. a.	42	46	55
Plt (× 10^9^/l)	50	n. a.	n. a.	71	51	n. a.	n. a.	86	n. a.	46	42	42
Fibrinogen (mg/dl)	218	n. a.	n. a.	288	255	n. a.	n. a.	197	n. a.	n. a.	n. a.	244
Hemoglobin (g/dl)	10.8	9.3	9.1	9.7	9.1	8.8	7.6	8.1	8.2	8.0	8.0	8.6
RBC transfused (U)	0	1	1	0	2	2	1	1	1	2	0	0

*aPTT* activated partial thromboplastin time; *CT* clotting time; *FVIII* factor VIII; *InTEM* intrinsic thromboelastometry; *IU* international unit; *n. a.* not available, *OLT* orthotopic liver transplantation; *Plt* platelets; *PT* prothrombin time, *RBC *red blood cell, † during reperfusion and signs of bleeding

During the entire perioperative and operative phase (last dosage of 4000 IE efmoroctocog alfa on the ICU), a total of 28,000 IU efmoroctocog alfa was needed. Additionally, 40 IU of FFP (10 l), 8 IU of RBC (2.4 l), 4 g fibrinogen and 4 IU platelets (1.2 l) were administered perioperatively. Signs of hemorrhage or other complications were not detected. At 24 h after surgery the patient was treated with additional 4000 IU of recombinant FVIII at the intensive care unit (ICU). As a result of the successful liver transplantation, no further medication with efmoroctocog alfa was required, because the grafted liver started endogenous FVIII production and constant hemostasis with physiological levels between 61–109% were maintained. The postoperative period proceeded without any bleeding events or complications. Additionally, no further surgical intervention was necessary and the patient left the ICU 4 days post-surgery.

## Discussion

Hemophilia A is an inherited insufficiency of FVIII. The gold standard for the management of moderate to severe hemophilia A is prophylaxis using regular replacement therapy with coagulation factor concentrates. In contrast to conventional treatment, extended half-life products reduce the frequency of factor replacement injections. A biphasic monoclonal antibody (Emicizumab; Roche, Portugal) mimicking activated FVIII, has also been approved for patients with hemophilia A with/without inhibitors. Further, gene therapy is an emerging therapy for hemophilia A. Although the efficacy and safety of extended half-life FVIII, such as efmoroctocog alfa in subjects requiring surgery have been demonstrated before [4], most of available data were obtained in the frame of clinical trials enrolling highly selected patients. Experience with the use of extended half-life FVIII products for bleeding management in the operational setting relates primarily to orthopedic interventions, which are often necessary in hemophilia patients due to bleeding events into the joints. In the present case report, the perioperative coagulation management in the real-world situation with an extended half-life FVIII product (efmoroctocog alfa) during major surgery associated with a high risk of bleeding in a patient suffering from severe hemophilia A is described.

Despite the fact that overall peri- and posttransplantation complication rates and long-term survival are about the same in hemophilic and non-hemophilic patients to date, the risk of bleeding during liver transplantation remains tremendous [1, 6]. We choose to guide efmoroctocog alfa perioperatively based on thromboelastometry InTEM (clotting time), as no 24/7 FVIII measurements are available at our institution and due to the advantage of a short turnaround time using thromboelastometry. Although the clotting time allowed us to estimate FVIII activity perioperatively, the patient received slightly more efmoroctocog alfa than indicated by FVIII measurements. The inaccuracy of the CT InTEM for guiding efmoroctocog alfa therapy is also attributed to the influence of other coagulation factors. The use of CT InTEM for guiding efmoroctocog alfa must be individually adapted to each patient based on individual pharmacokinetic data. Due to the risk of overdosing, this method can therefore not be generally recommended; however, the use of efmoroctocog alfa administered as bolus injections was efficacious and safe allowing a perioperative bleeding management taking into account the patient’s individual pharmacokinetic profile. Hemostatic control was achieved and bleeding was minimized leading to a successful transplantation. Thereby, a total of 24,000 IU efmoroctocog alfa were sufficient to keep stable FVIII levels above 100% during the perioperative phase on the day of surgery until the postoperative day. After the initial supply of 8000 IU, 4 repetitive doses of 4000 IU were given. Due to the extended half-life, further supply was necessary only once 24 h postsurgery. The consumption data are within the range of other data published for HCV-infected hemophilia A patients undergoing liver transplantation [7].

In the presented case, treatment with an extended half-life FVIII was well tolerated without any side effects. These results support the efficacy and safety results known from other studies performed with extended half-life FVIII products [3]. Furthermore, our findings are consistent with those in another case report describing a successful perioperative bleeding management with efmoroctocog alfa in a patient with severe hemophilia A and central venous catheter replacement [2]; however, it has to be taken into account that in contrast to the presented case efmoroctocog alfa was substituted as continuous infusion and not as bolus in an infant within a different operational setting. In general, direct comparisons with surgical studies in hemophilia A patients are hardly feasible. This may be due to different surgical methodologies and FVIII administration modalities, the limited number and high variability of persons affected. Analyses of combined data derived from three studies performed with efmoroctocog alfa indicate consumption levels during major surgery similar or even advantageous to standard recombinant FVIII products [4]; however, more investigations are a prerequisite to analyze a possible lowering of the overall clotting factor consumption and thus related pharmacoeconomic consequences.

## Conclusion

The present case demonstrates that the use of an extended half-life FVIII is suitable for a successful perioperative bleeding control even in hemophilia patients at high bleeding risk during major surgery. Due to the EHL constant FVIII levels could be reached with relatively few injections. In order to confirm the obtained results, more real-world data in different operative settings are essential.

## Declarations

For this article no studies with human participants or animals were performed by any of the authors. Written informed consent was obtained from the patient.

The supplement containing this article is not sponsored by industry.
